# Prostate cancer outcome in Burkina Faso

**DOI:** 10.1186/1750-9378-6-S2-S6

**Published:** 2011-09-23

**Authors:** Fasnéwindé A Kabore, Barnabé Zango, Adama Sanou, Clotaire Yameogo, Brahima Kirakoya

**Affiliations:** 1 Division of urology, Yalgado Ouédraogo University hospital of Ouagadougou 03 Po Box 7022, Burkina Faso; 2 Division of general surgery Yalgado Ouédraogo University hospital of Ouagadougou 03 Po Box 7022, Burkina Faso

## Abstract

**Introduction:**

African-American black men race is one of non-modifiable risk factors confirmed for prostate cancer. Many studies have been done in USA among African- American population to evaluate prostate cancer disparities. Compared to the USA very few data are available for prostate cancer in Sub-Saharan African countries. The objective of this study was to describe incident prostate cancer (PC) diagnosis characteristics in Burkina Faso (West Africa).

**Methods:**

We performed a prospective non randomized patient’s cohort study of new prostate cancer cases diagnosed by histological analysis of transrectal prostate biopsies in Burkina Faso. Study participants included 166 patients recruited at the urology division of the university hospital of Ouagadougou. Age of the patients, clinical symptoms, digital rectal examination (DRE) result, serum prostate-specific antigen (PSA) level, histological characteristics and TNM classification were taking in account in this study.

**Results:**

166 transrectal prostate biopsies (TRPB) were performed based on high PSA level or abnormal DRE. The prostate cancer rate on those TRPB was 63, 8 % (n=106). The mean age of the patients was 71, 5 years (52 to 86). Urinary retention was the first clinical patterns of reference in our institution (55, 7 %, n = 59). Most patients, 56, 6 % (n = 60) had a serum PSA level over than 100 ng/ml. All the patients had adenocarcinoma on histological study of prostate biopsy cores. The majority of cases (54, 7 % n = 58) had Gleason score equal or higher than 7.

**Conclusion:**

Prostate cancer is diagnosed at later stages in our country. Very high serum PSA level and poorly differentiated tumors are the two major characteristics of PC at the time of diagnosis.

## Introduction

African-American black men race is one of non-modifiable risk factors confirmed for prostate cancer (PC). Many studies have been done in USA among African-American to evaluate prostate cancer disparities in this population. African-Americans have more advanced stages of PC at diagnosis and higher mortality rates for PC than whites. [1-4] There is no scientific proof that PC in African-Americans has the same characteristics in their ancestry from Central and West Africa but there is documented evidence in the literature indicating that prostate cancer in one West African country, Nigeria, is similar to rates found in the United States [5]. Compared to the USA very few data are available for prostate cancer in Sub-Saharan African countries [1,2]. This work aims to contribute by providing more data about PC in West Africa and to participate in the research to reduce prostate cancer disparities in black men. In Burkina Faso (West Africa) PC epidemiology, clinical and pathological characteristics are not well evaluated. This study is the first to assess PC particularity at the biggest urology division in the university hospital of the main city of Burkina Faso: Ouagadougou. The objective of the study was to describe PC diagnosis characteristics at the time of diagnosis in our country.

## Methods

We performed a prospective non randomized patient’s cohort study of new PC cases diagnosed in Burkina Faso. Study participants included 166 patients recruited at the urology division of the university hospital of Ouagadougou. Our institution receives patients from all over the country. PC cases were diagnosed at histological analysis of transrectal prostate biopsy (TRPB) core among 166 patients who had abnormal digital rectal examination (DRE) or high serum prostate-specific antigen (PSA) level. The recruitment of all patients was made between January 2009 and June 2010. The variables of interest included age at diagnosis, the clinical symptoms of reference in our center for PC, serum PSA level, information about prostate biopsy (histological type, Gleason score) and stage of tumor determined according to the TNM 2007 classification. The existence of metastatic PC was evaluated with clinical exam and thoraco-abdominal and pelvis computed tomography. Descriptive statistics were used to summarize these variables in terms of the means, extremes values, and frequencies.

## Results

**Figure 1:**
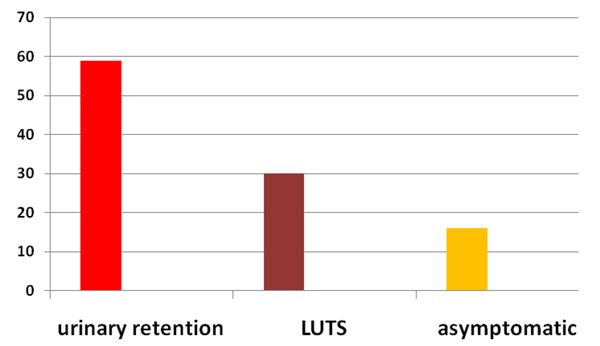
Repartition of patients according to Clinical symptoms

**Figure 2:**
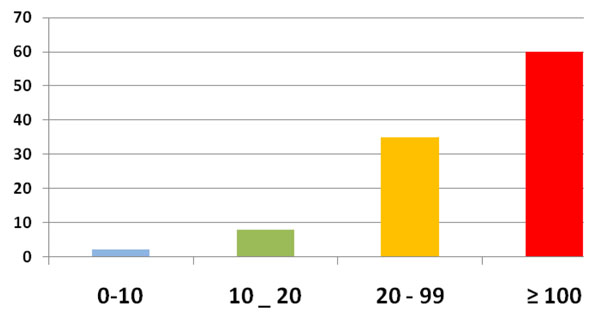
Repartition of patients according to Serum PSA level

**Figure 3:**
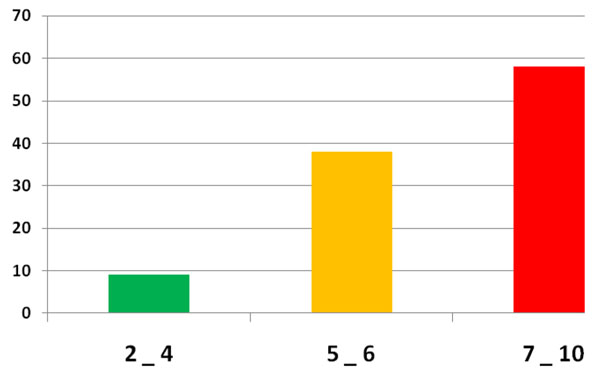
Repartition of patients according to Gleason score

One hundred and sixty six (166) TRPB were performed based on high PSA level or abnormal DRE. The prostate cancer rate on those TRPB was 63, 8 % (n =106). The mean age of the patients was 71, 5 years (52 to 86). All patients had abnormal DRE. Figure 1 summarizes the clinical symptoms at the time of diagnosis. The mean duration of symptoms prior to presentation was 12.6 months (range: 1 month to 6 years). Urinary retention was the first clinical patterns of reference in our institution (55, 7 %, n = 59). Most patients, 56, 6% (n = 60) had a serum PSA level over than 100 ng/ml [figure 2]. The mean serum PSA level was 537ng/ml (8, 41 to 17850). All the patients had adenocarcinoma on histological study of prostate biopsy cores. The majority of cases (54, 7 % n = 58) had Gleason score equal or higher than 7 [Figure 3]. Most patients, 73, 6 % (n = 78) had their primary tumor extended over the prostatic capsule (T3-T4 stages). Table 1 summarizes the lymph nodes (N) involvement and metastatic PC (M) according to primary tumor stage (T).

**Table 1:** Lymph nodes involvement and metastatics localizations according to primary tumor stage

Primary tumor (T)	n/%	Lymph node Involvement (N) n/%	Metastatic Localization (M) n/%
**T1**	0/0	0/0	0/0
**T2**	28/26,4	3/2,8	0/0
**T3**	40/37,7	12/11,3	12/11,3
**T4**	38/35,9	30/28,3	32/30,2

**Total**	**106/100**	**45/42,4**	**44/41,5**

## Discussion

Numerous studies have reported that black men are diagnosed with prostate cancer at a younger age [3,6] than white men. Many other studies suggest that there are no age differences at the time of PC diagnosis [4,7]. In our study the mean age is higher (71, 5). Older age at the time of diagnosis of PC is commonly reported from West Africa [8-11]. The majority of patients in this study (84 %) presented urinary retention or lower urinary tract symptoms (LUTS). According to Ajapé [8] the severity of urinary symptoms at the time of PC diagnosis in low resources countries is relevant to late presentation, ignorance of the population concerning PC symptoms, and poverty. Furthermore, the lack of urology centers (only two public urology centers in Burkina Faso) can be a reason of late presentation.

Studies have reported that African-American men diagnosed with prostate cancer have greater PSA levels than whites [3,9,12,13]. The results of our study strengthen these reports. However the mean PSA level in our study is very high. This is relevant to late presentation and the fact that several patients included in this study were empirically managed for several years for PC without PSA testing or biopsy in poorly equipped rural centers. However we cannot exclude a possible “racial-ethnical” particularity in our country but this must be assessed by further studies. The relation between high PSA level in black men, race and genetic is not well established. Veda [12] finds no evidence in support of the traditional “race-specific” PSA. He reports that the PSA has a higher prediction for PC in African-American men but concludes that this finding may be explained by genetic West African ancestry which needs further study.

Most PC cases reported in our study were high grade tumors with poor prognosis (PSA > 20 ng/ml, Gleason score 7-10, or clinical stage T3-T4). The explanation is late diagnosis and the lack of PC screening program in our country. The Gleason score is the most reliable factor in determining the biological characteristics of prostate cancer, tumor stage, and its prognosis. [3,14] Several reports that black men presented with more poorly differentiated PC than white. [2,3,15] These results suggest more studies to evaluate if other factors such as diet, lifestyle, genetics played a role in high Gleason score reported in black men prior to late diagnosis and limited access to medical center.

PC screening remains controversial and expose to over diagnosis and overtreatment. In a recent review, Stavridis [16] concludes that, as we are aware, there is no sufficient evidence to recommend routine population screening for PC. Nevertheless, clinicians should provide information about potential benefits and risks of screening for PC, and the limitations of current evidence for screening, and make the final decision to screen or not. However, screening has the potential to reduce PC disparities in African black men. A good PC screening policy in Burkina Faso can reduce specific mortality by early diagnosis and raise the population awareness of PC. Arthur [17] in Ghana promotes screening to improve early detection, management and prognosis of PC.

## Conclusion

PC is diagnosed at later stages in Burkina Faso. Very high serum PSA level and poorly differentiated tumors are the two major characteristics of PC at the time of diagnosis. Interventions focused on screening, education about the disease and early detection could substantially improve these findings. This work suggests more studies are needed in this country to better access PC epidemiology, genetics, mortality and survival particularities.

## Competing interests

No competing interests.

## Authors' contributions

FAK conceptualized, designed the study and wrote the paper. ZB, CY, AS and BK participated in data collection and reviewed the manuscript. All the authors reviewed and approved the manuscript.
